# CD4 criteria improves the sensitivity of a clinical algorithm developed to identify viral failure in HIV-positive patients on antiretroviral therapy

**DOI:** 10.7448/IAS.17.1.19139

**Published:** 2014-09-15

**Authors:** Denise H Evans, Matthew P Fox, Mhairi Maskew, Lynne McNamara, Patrick MacPhail, Christopher Mathews, Ian Sanne

**Affiliations:** 1 Health Economics and Epidemiology Research Unit, Department of Internal Medicine, School of Clinical Medicine, Faculty of Health Sciences, University of the Witwatersrand, Johannesburg, South Africa; 2 Boston University, Center for Global Health & Development, Boston, MA, USA; 3 Clinical HIV Research Unit, Department of Internal Medicine, School of Clinical Medicine, Faculty of Health Sciences, University of the Witwatersrand, Johannesburg, South Africa; 4 Right to Care, Johannesburg, South Africa; 5 Department of Medicine, University of California, San Diego, CA, USA

**Keywords:** antiretroviral therapy, viral load, resource limited, monitoring, algorithm, HIV, CD4

## Abstract

**Introduction:**

Several studies from resource-limited settings have demonstrated that clinical and immunologic criteria are poor predictors of virologic failure, confirming the need for viral load monitoring or at least an algorithm to target viral load testing. We used data from an electronic patient management system to develop an algorithm to identify patients at risk of viral failure using a combination of accessible and inexpensive markers.

**Methods:**

We analyzed data from HIV-positive adults initiated on antiretroviral therapy (ART) in Johannesburg, South Africa, between April 2004 and February 2010. Viral failure was defined as ≥2 consecutive HIV-RNA viral loads >400 copies/ml following suppression ≤400 copies/ml. We used Cox-proportional hazards models to calculate hazard ratios (HR) and 95% confidence intervals (CI). Weights for each predictor associated with virologic failure were created as the sum of the natural logarithm of the adjusted HR and dichotomized with the optimal cut-off at the point with the highest sensitivity and specificity (i.e. ≤4 vs. >4). We assessed the diagnostic accuracy of predictor scores cut-offs, with and without CD4 criteria (CD4 <100 cells/mm^3^; CD4 < baseline; >30% drop in CD4), by calculating the proportion with the outcome and the observed sensitivity, specificity, positive and negative predictive value of the predictor score compared to the gold standard of virologic failure.

**Results:**

We matched 919 patients with virologic failure (1:3) to 2756 patients without. Our predictor score included variables at ART initiation (i.e. gender, age, CD4 count <100 cells/mm^3^, WHO stage III/IV and albumin) and laboratory and clinical follow-up data (drop in haemoglobin, mean cell volume (MCV) <100 fl, CD4 count <200 cells/mm^3^, new or recurrent WHO stage III/IV condition, diagnosis of new condition or symptom and regimen change). Overall, 51.4% had a score 51.4% had a score ≥4 and 48.6% had a score <4. A predictor score including CD4 criteria performed better than a score without CD4 criteria and better than WHO clinico-immunological criteria or WHO clinical staging to predict virologic failure (sensitivity 57.1% vs. 40.9%, 25.2% and 20.9%, respectively).

**Conclusions:**

Predictor scores or risk categories, with CD4 criteria, could be used to identify patients at risk of virologic failure in resource-limited settings so that these patients may be targeted for focused interventions to improve HIV treatment outcomes.

## Introduction

In 2012, 9.7 million people in low- and middle-income countries received antiretroviral therapy (ART), representing 61% of all who were eligible under the 2010 WHO HIV treatment guidelines [1]. When patients start ART, mortality is highest during the first months of therapy [2]. This high-risk period requires careful monitoring of patients for disease progression, potential drug toxicity and treatment failure. As more people in resource-limited settings receive ART, treatment failure and the need to switch drug regimens will increase [3].

Measurement of HIV viral RNA (viral load) has been shown to be one of the best predictors of clinical disease progression, as well as being the main parameter to assess treatment response in HIV-positive patients [4]. The diagnosis of treatment failure in many resource-constrained settings is difficult because of limited access to plasma HIV-RNA testing and because few laboratories can afford to perform these tests routinely [5–9]. Not only are the laboratory assays (e.g. CD4 cell counts and HIV-RNA testing) for monitoring treatment expensive, but they are technically demanding, requiring high levels of expertise and equipment – thereby limiting widespread use in resource-poor settings [10]. Monitoring of ART with dual HIV-RNA and CD4 count testing is often unsustainable within national health programmes in resource-constrained settings. Currently, there is no real alternative to CD4 count testing for monitoring immunologic responses to treatment [10]. The World Health Organization (WHO) recommends clinico-immunological criteria to identify first-line treatment failure in resource-constrained settings; however, this has limited sensitivity and positive predictive value which can lead to both delayed and inappropriate premature switching to more expensive second-line agents [11–15]. Several studies from resource-limited settings have demonstrated that clinical and immunologic criteria are poor predictors of virologic failure and, therefore, the need for a viral load monitoring algorithm exists [16,17]. Results of work on predictive markers has been conflicting [7,9,10,18,19]. Thus, the need for low-cost surrogate markers of virologic failure that are widely available, even in resource-limited settings, is essential. Numerous attempts at identifying such markers have been made with the intent of alleviating the need for frequent CD4 count or viral load testing [10]. For example, Meya and colleagues reported that an algorithm including data on patient adherence to drug regimens, CD4 cell count and clinical criteria may identify those at risk for virologic failure and this can be used to allocate viral load testing in resource-limited settings more efficiently and cost-effectively [6].

With the expansion of ART, there is an urgent need for alternative low-cost predictors of virologic failure in resource-limited settings. Monitoring the efficacy of ART in resource-constrained settings remains a critical challenge and current research priorities include optimizing monitoring strategies and developing simpler, cheaper assays that can be administered by minimally trained clinic personnel [5]. Targeted viral load testing in a subgroup of patients with an increased risk of treatment failure may be a feasible and effective strategy for resource-limited settings. As the South African guidelines for monitoring HIV-patients on ART require regular CD4 counts and viral load testing [20–22], we set out to use data from routine care in a large-volume ART clinic to identify low-cost predictors of treatment failure in order to identify patients in need of targeted viral load testing.

## Methods

### Study site and subjects

The study was a retrospective analysis of data collected from a cohort of HIV-positive adults initiated on ART at the Themba Lethu Clinic (TLC), Johannesburg. This cohort has been described elsewhere [23]. By the end of 2011, TLC has provided care for over 29,000 patients with HIV, including 20,600 on ART. Patients at Themba Lethu Clinic are initiated on ART and receive routine follow-up visits according to South African National Department of Health ART treatment guidelines [20–22]. Longitudinal, clinical and demographic data are collected and stored on an electronic patient management system, TherapyEdge-HIV (Associated Biological Systems, South Africa), and exported into SAS 9.1 (SAS Institute Inc., Cary, NC) for the analysis. This study was approved by the Human Research Ethics Committee of the University of the Witwatersrand.

### Inclusion criteria

Eligible subjects included HIV-positive ART naïve adults (≥18 years of age) who were initiated according to South African National Department of Health ART treatment guidelines onto a standard first-line regimen of stavudine (d4T) or zidovudine (AZT) with lamivudine (3TC) and either efavirenz (EFV) or nevirapine (NVP) between April 2004 and February 2010. The analysis was limited to patients who had two or more viral load results recorded and more than six months on ART. We excluded those who were not ART naïve, those who transferred in from another antiretroviral (ARV) treatment site, those with a baseline CD4 count >350 cells/mm^3^, those with baseline viral load ≤400 copies/ml and those receiving second-line ART (regimen containing either didanosine or a protease inhibitor such as lopinavir-ritonavir) at ART initiation. We also excluded pregnant women as they are initiated using different criteria and are typically placed on lopinavir and ritonavir rather than EFV.

### Study variables

Since 2004, patient data have been captured electronically. Between 2004 and 2010, monitoring tests were done every six months but after 2010 this changed to 6 and 12 months during the first year of treatment and yearly thereafter [23]. After ART initiation, ARV drug collection visits are scheduled monthly for the first 6–12 months on treatment and every two months thereafter once stable whereas medical follow-up visits are scheduled at months one, three, six, and twelve thereafter depending on the regimen.

Our primary outcome was virologic failure defined as sustained or two consecutive HIV-RNA (≥400 copies/ml) following suppression below this level [24]. Viral failure was assessed at three time points: 1) ever, 2) in the first 18 months after ART initiation and 3) after the first 18 months. We developed the monitoring algorithm in patients with virologic failure and time-matched controls (±1 month) who did not fail, using greedy matching (1:3).

Candidate predictors for the monitoring algorithm were divided into baseline and follow-up variables. Over 27 inexpensive, routinely collected variables were included in a preliminary analysis [25,26]. These included body mass index (BMI; weight in kg/height in m^2^), haemoglobin (Hb), haematocrit (%), to mean corpuscular haemoglobin (MCH), mean corpuscular haemoglobin concentration (MCHC), mean cell volume (MCV), platelet count, total lymphocyte count, white blood cell (WBC) count, systolic and diastolic blood pressure, oral candidiasis, WHO stage III or IV condition and serum albumin. Of the 27, 12 were included in the final predictor score.

Follow-up variables were defined as 1) the clinical and laboratory parameter at the visit prior to the outcome if the parameter was a single measurement (i.e. Hb <10 g/dl, MCV <100 fl, CD4 count <200 cells/mm^3^, current weight below baseline or new/recurrent WHO stage III/IV condition) and 2) the difference in value between the two visits prior to the outcome (i.e. drop in MCV or haematocrit by more than 20% or drop in weight by more than 5 kg). The cut-offs used for these variables were obtained from literature and a dichotomous outcome was obtained for each variable (0 for No and 1 for Yes) [5,6,18,27]. For other variables, including measures of adherence, missed medical visits or missed ARV drug pickups (defined as missing a scheduled visit by more than seven days), the (re)appearance or diagnosis of a new symptom or opportunistic infection and regimen change or substitution, if these were reported within 6–9 months prior to the outcome then the variable was dichotomized as Yes (1) Where there were no missed medical visits or missed ARV drug pickups, no (re)appearance or diagnosis of a new symptom or opportunistic infection and no regimen change during the 6–9 months prior to the outcome, the variable was dichotomized as No (0). While CD4 testing may be available in most settings, this is not the case in every country [28]. Therefore, two scenarios were considered: 1) including clinical factors alone and 2) including both clinical factors and CD4.

### Statistical analysis

Patient demographics and baseline characteristics were summarized using means with standard deviation for normally distributed variables, medians with interquartile range for not normally distributed variables and proportions for categorical variables. We assessed the association between the different variables or predictors and the risk of virologic failure using Cox-proportional hazard models to estimate the hazard ratio (HR) and 95% confidence interval (CI). Person time accrued from ART initiation until the earliest of 1) date of virologic failure, 2) date of death, 3) date of loss to follow-up (defined as missing their last scheduled visit by more than 122 days), 4) date of transfer or 5) administrative censoring (close of dataset). Proportional hazard assumptions were formally checked using log–log survival curves and goodness of fit tests for predictor variables.

We then developed a scoring system following the approach of Spiegelhalter and Knill-Jones [29,30]. We created a predictor score for each patient defined as the sum of the natural logarithm of the adjusted HR for each predictor rounded to the nearest integer [5]. The predictor score was dichotomized using different cut-offs ranging from ≥1 vs. <1 to ≥7 vs. <7. We assessed the diagnostic accuracy of each cut-off (i.e. ≥4 vs. <4) by calculating the proportion with the outcome and the observed sensitivity (Se), specificity (Sp), PPV and NPV of predictor scores compared to the gold standard of virologic failure. The optimal cut-off, as guided by Receiver operator curves (ROC) to calculate the area under curve (AUC), was identified as the point with the highest sensitivity and acceptable specificity. Logistic regression was used to estimate the odds ratio to determine associations between each cut-off and virologic failure.

## Results

Of a total of 11,582 patients that initiated ART, 32% (2632/8225) never achieved an undetectable viral load. A total of 3675 patients were included in the analysis, 919 with virologic failure (rebound after initial virologic suppression) matched (1:3) to 2756 patients who did not experience virologic failure during follow-up. Baseline demographics and clinical characteristics were similar between the two groups, although those with virologic failure appeared somewhat more immunosuppressed (Table 1).

**Table 1:** Demographic and clinical characteristics of HIV-positive patients with virologic failure (*n*=919) and time-matched controls (*n*=2756) at the Themba Lethu Clinic in Johannesburg, South Africa, between 2004 and 2010 (*n*=3675)

Baseline characteristics		Virologic failure (*n*=919)	No virologic failure (*n*=2756)
Sex – Male	n,%	342 (39.7%)	1095 (39.0%)
Age	Median (IQR)	34.7 (30.2–41.4)	36.2 (31.1–42.8)
Body mass index (kg/m^2^)	Median (IQR)	21.4 (18.9–24.6)	21.3 (18.8–24.3)
< 18.5 kg/m^2^	n,%	177/856 (20.7%)	544/2515 (21.6%)
CD4 cell count (cells/mm^3^)	Median (IQR)	65 (23–130)	89 (34–154)
< 50 cells/mm^3^	n,%	339/831 (40.8%)	841/2530 (33.2%)
51–100 cells/mm^3^	n,%	209/831 (25.1%)	533/2530 (21.1%)
101–200 cells/mm^3^	n,%	241/831 (29.0%)	945/2530 (37.4%)
> 200 cells/mm^3^	n,%	42/831 (5.1%)	211/2530 (8.3%)
Haemoglobin (g/dl)	Median (IQR)	11.4 (10.0–12.9)	11.5 (10.0–13.0)
< 8 g/dl	n,%	51/852 (6.0%)	165/2611 (6.3%)
Viral load >100,000 copies/ml	n,%	98/194 (50.5%)	280/687 (40.8%)
WHO stage III/IV	n,%	385/830 (46.4%)	1030/2493 (41.3%)
First regimen d4T-3TC-EFV	n,%	778 (84.7%)	2408 (87.4%)
d4T-3TC-NVP	n,%	94 (10.2%)	185 (6.7%)
Other	n,%	47 (5.1%)	163 (5.9%)
Tuberculosis at ART initiation	n,%	171 (18.6%)	438 (15.9%)
Time on ART (months)	Median (IQR)	26.4 (15.4–42.2)	26.2 (15.4–42.1)

BMI, body mass index; WHO, World Health Organization; d4T, stavudine; 3TC, lamivudine; EFV, efavirenz; NVP, nevirapine.

In crude analysis, numerous baseline and follow-up variables were associated (*p*<0.1) with virologic failure. These variables were entered into a multivariate Cox-proportional hazards model and characteristics that maintained statistical significance are shown in Table 2. Twelve variables were included in the final predictor score. We adjusted for gender in the Cox-proportional hazards model although this variable did not contribute to the overall score. The predictor score included ART initiation variables (age, CD4 count <100 cells/mm^3^, WHO stage III/IV and albumin <25 g/l) and laboratory and clinical follow-up data such as a drop in haemoglobin, MCV <100 fl, CD4 count <200 cells/mm^3^, new or recurrent WHO stage III/IV condition, diagnosis of new condition or symptom and regimen change. Only new or recurrent WHO stage III/IV condition had a score of +2 while all the other variables had a score of +1. In agreement with other scoring systems, this predictor score also included adherence, measured by the number of missed medical or ARV drug pickup, as other measures (i.e. self-reported adherence) had too few observations to assess this tool [6,31].

**Table 2:** Crude and adjusted predictors of virologic failure comparing HIV-positive patients with virologic failure (*n*=919) to controls on ART for an equal duration but without virologic failure (*n*=2756) at the Themba Lethu Clinic in Johannesburg, South Africa, between 2004 and 2010

	% with virologic failure (*n*=919)	Crude HR 95% CI	Adjusted HR 95% CI	Score^a^
Baseline – at ART initiation				
Sex				
Female	577 (62.8%)	Reference	Reference	+0
Male	342 (37.2%)	0.97 (0.85–1.11)	0.95 (0.77–1.15)	+0
Age at ART initiation				
≤ 40 years	274 (29.8%)	Reference	Reference	+0
> 40 years	645 (70.2%)	1.16 (1.03–1.33)	1.30 (1.06–1.60)	+1
CD4 cell count				
≥ 100 cells/mm^3^	283/831 (34.1%)	Reference	Reference	+0
< 100 cells/mm^3^	548/831 (65.9%)	1.35 (1.17–1.56)	1.20 (1.09–1.48)	+1
WHO stage				
I/II	445/830 (53.6%)	Reference	Reference	+0
III/IV	385/830 (46.4%)	1.16 (1.01–1.33)	1.26 (1.02–1.57)	+1
Albumin				
≥ 25 g/l	617/788 (78.3%)	Reference	Reference	+0
< 25 g/l	171/788 (21.7%)	1.20 (1.01–1.42)	1.15 (0.95–1.45)	+1
Follow-up variables (6–12 months prior to failure)				
Haemoglobin drop				
≤ 20%	801/823 (97.3%)	Reference	Reference	+0
> 20%	22/823 (2.7%)	1.60 (1.05–2.45)	1.38 (0.97–2.77)	+1
Mean cell volume (MCV)				
≥ 100 fl	386/775 (49.8%)	Reference	Reference	+0
< 100 fl	389/775 (50.2%)	1.29 (1.12–1.49)	1.23 (1.02–1.49)	+1
CD4 cell count				
≥ 200 cells/mm^3^	536/821 (65.3%)	Reference	Reference	+0
< 200 cells/mm^3^	285/821 (34.7%)	1.37 (1.19–1.59)	1.28 (1.02–1.60)	+1
Missed a medical or ARV drug pickup >7 days				
No	718/816 (87.9%)	Reference	Reference	+0
Yes	98/816 (12.1%)	1.48 (1.22–1.79)	1.26 (0.97–1.64)	+1
New condition/diagnosis				
No	609/770 (79.1%)	Reference	Reference	+0
Yes	161/770 (20.9%)	1.34 (1.16–1.55)	1.14 (0.97–2.07)	+1
Regimen change/substitution				
No	698/919 (76.0%)	Reference	Reference	+0
Yes	221/919 (24.0%)	1.25 (1.08–1.46)	1.26 (1.01–1.57)	+1
New or recurrent WHO stage				
I or II	702/737 (95.3%)	Reference	Reference	+0
III or IV	35/737 (4.8%)	1.52 (1.08–2.13)	1.58 (1.03–2.46)	+2

a A score was calculated as the sum of the natural logarithm of the adjusted hazard ratios for each predictor rounded to the nearest integer.

WHO, World Health Organization; ARV, antiretroviral; ART, antiretroviral therapy, HR, Hazard Ratio; CI, confidence interval.

The optimal cut-off of four was selected for the predictor score (≥4 vs. <4 Se 57.1%; Sp 50.5%). A cut-off ≥5 vs. <5 had a Se of 37.3% and Sp of 66.3%, while a cut-off of ≥3 vs. <3 had a Se of 75.7% and Sp of 32.9%. About half (51.4%) of all patients had a risk score ≥4 while the remainder (48.6%) had a risk score <4. Virologic failure was more common among those with a risk score ≥4 (27.8%, 525/1889) compared to patients with risk score <4 (22.1%, 394/1786). Compared to patients with a predictor score <4, patients with a predictor score ≥4 were more likely to fail virologically (odds ratio 1.36 95% CI 1.17–1.59). Patients with a predictor score ≥2 had a higher odds of virologic failure (OR 1.61 95% CI 1.31–1.98) compared to those with a score of <2, although the cut-off would result in more false positives compared to those with a cut-off ≥4 (Sp 14.8% vs. 50.5%). The AUC of a predictor score ≥4 (including information about CD4 count) to correctly identify virologic failure was calculated as 0.62.

**Figure 1:**
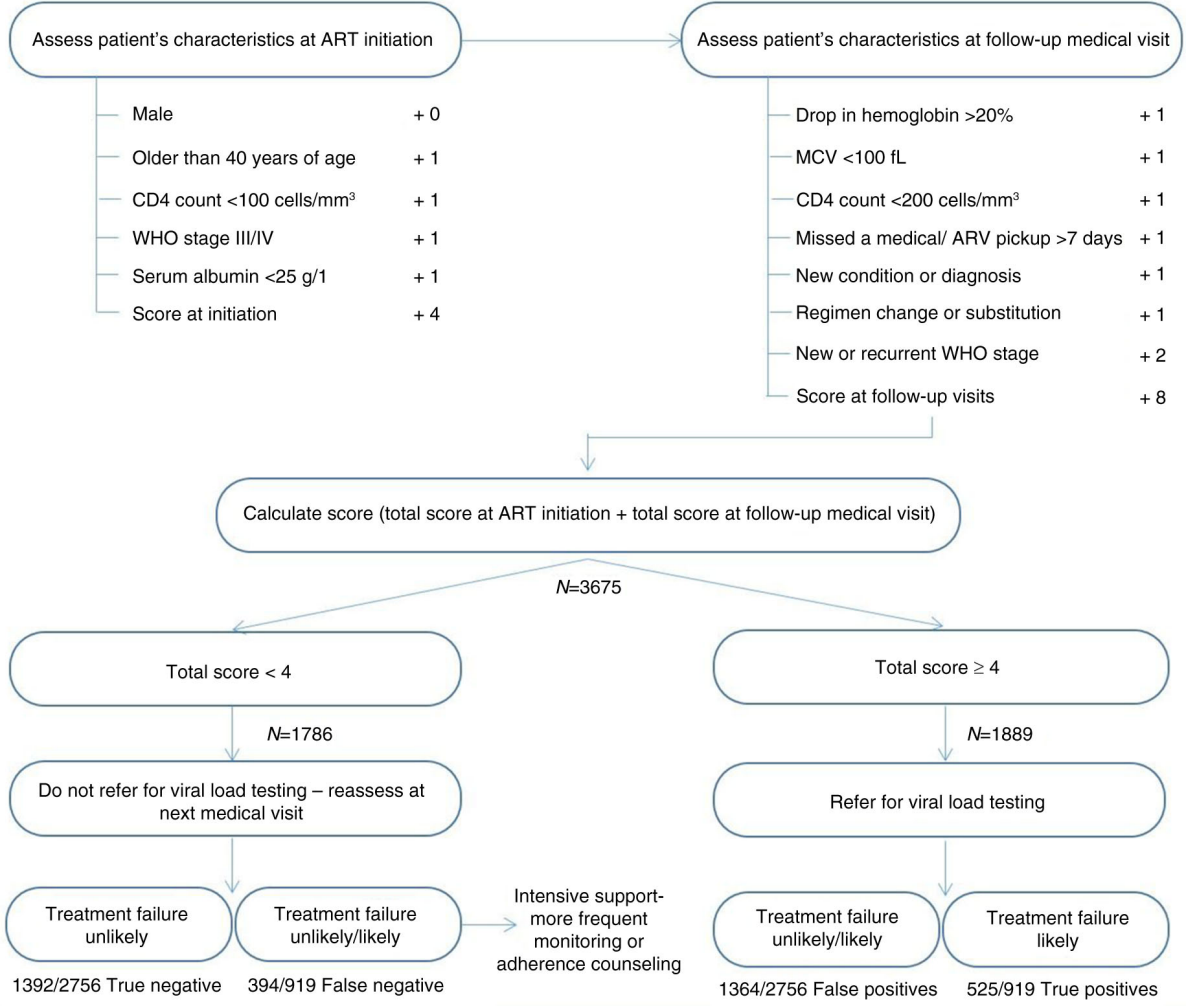
Algorithm for targeted viral load testing using CD4 criteria. Baseline (*n*=5) and follow-up (*n*=7) variables are used to calculate the total score. The diagram shows how a cut-off score of <4 and ≥4 can be used to manage patients at low-risk (reassess at next medical visit) or medium to high-risk (refer for viral load testing) for virologic failure.

**Figure 2:**
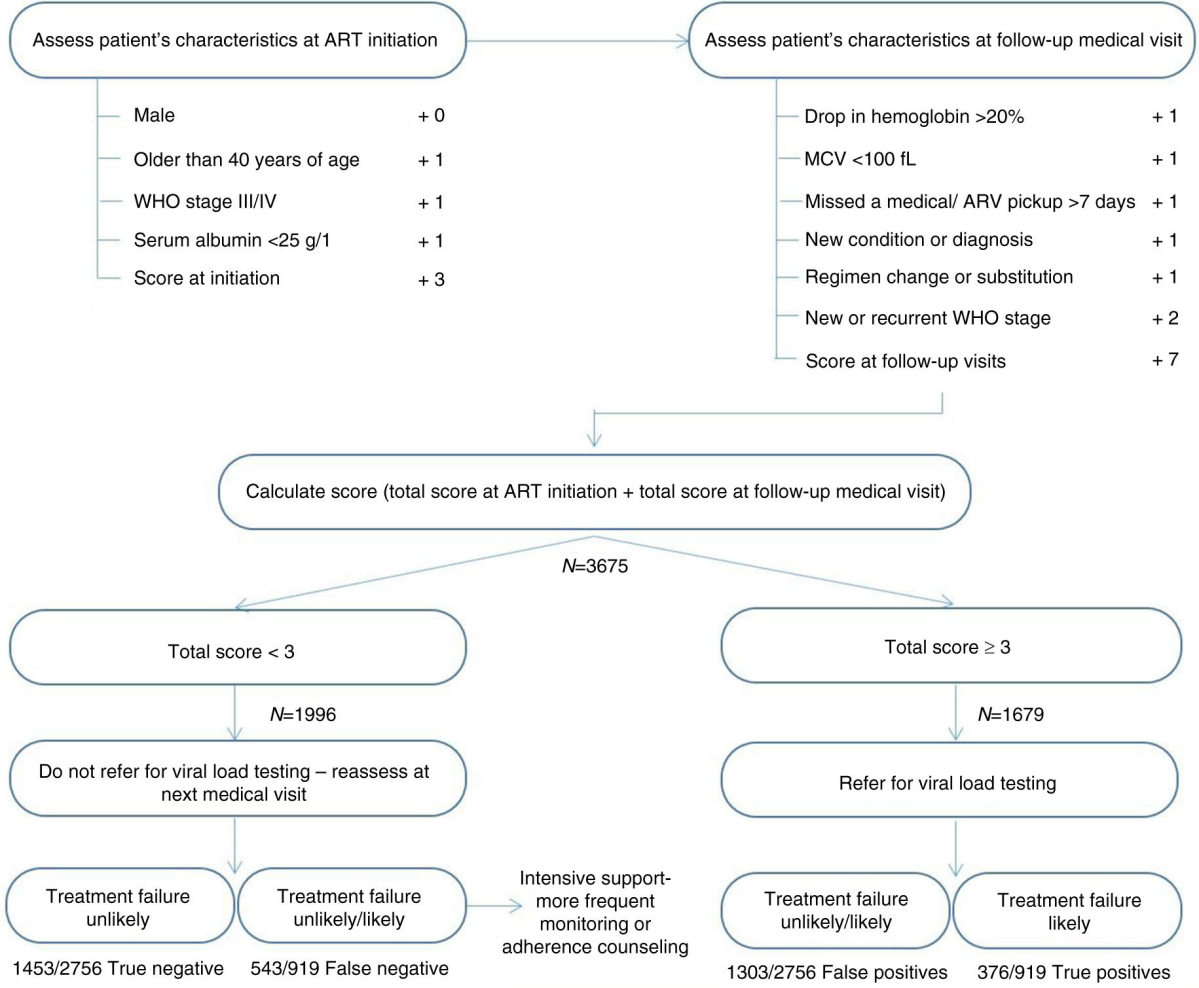
Algorithm for targeted viral load testing without CD4 criteria. Baseline (*n*=4) and follow-up (*n*=6) variables are used to calculate the total score. The diagram shows how a cut-off score of <3 and ≥3 can be used to manage patients at low-risk (reassess at next medical visit) or medium to high-risk (refer for viral load testing) for virologic failure.

We assessed the diagnostic accuracy of the total predictor score to identify virologic failure, defined by viral load, as the gold standard. The sensitivity and specificity of the total predictor score (including CD4 criteria) was 57.1% and 50.5%, respectively (Figure 1). Using the same cut-off as that of the algorithm with CD4 criteria (≥4 vs. <4), the sensitivity increased to 62.0% but the specificity decreased to 34.6% when we ignored the information about patient CD4 count. When using a cut-off of ≥3 vs. <3, the sensitivity was 40.9% and the specificity was 52.7% while the sensitivity and specificity of a cut-off of ≥5 vs. <5 was 78.6% and 20.7%, respectively (Figure 2).

When virologic failure was stratified by time on ART, that is, 1) virologic failure in the first 18 months after ART initiation and 2) virologic failure after 18 months on ART, the sensitivity decreased from 67.3% to 51.8% while the specificity increased from 41.7% to 55.1%, respectively (Table 3). When restricting the analysis to patients on second-line ART at time of virologic failure, (220/919; 23.9%), the sensitivity increased to 58.6%, the specificity remained at 50.7% and the PPV improved from 27.8% to 41.0%.

**Table 3:** Diagnostic accuracy of predictor score with and without CD4 criteria and WHO criteria to identify virologic failure in patients at the Themba Lethu Clinic in Johannesburg, South Africa, between 2004 and 2010

Score (using a cut-off of ≥4 vs. <4)	Se	Sp	PPV	NPV
Total Score with CD4 criteria	57.1 (53.9–60.4)	50.5 (48.6–52.4)	27.8 (25.8–29.9)	77.9 (75.9–79.8)
Score without CD4 criteria (using a cut-off of ≥3 vs. <3)	40.9 (37.8–44.2)	52.7 (50.8–54.6)	22.4 (20.4–24.4)	72.8 (70.8–74.7)
Total Score (≤18 months)	67.3 (62.5–71.9)	41.7 (40.1–43.2)	27.7 (25.7–29.6)	79.3 (76.3–82.2)
Total Score (>18 months)	51.8 (48.3–55.3)	55.1 (54.0–56.3)	27.8 (25.9–29.7)	77.4 (75.8–79.1)
Clinical and immunological WHO criteria^a^	25.2 (22.8–27.8)	70.6 (69.8–71.5)	22.3 (20.1–24.6)	73.9 (73.1–74.8)
WHO stage III/IV	20.9 (18.4–23.6)	73.7 (72.8–74.6)	21.7 (19.1–24.4)	72.8 (72.0–73.7)
Total score in patients failing second-line ART^b^	58.6 (52.0–64.9)	50.7 (45.6–55.7)	41.0 (35.7–46.5)	67.7 (62.1–72.9)

a Defined as new or recurrent WHO stage III/IV, CD4 count below baseline or CD4 <100 cells/mm^3^ [5,6].

b Defined as a protease inhibitor-based (lopinavir-ritonavir) regimen with ≥1 change in nucleoside reverse transcriptase inhibitor.

Se, Sensitivity; Sp, Specificity; PPV, Positive predictive value; NPV, Negative predictive value.

We included two definitions of the WHO criteria for treatment failure in a sensitivity analysis, that is, 1) clinical and immunological WHO criteria which included a new or recurrent stage 3 or 4 condition, a CD4 count below baseline and persistent CD4 count below 100 cells/mm^3^ and 2) new or recurrent stage 3 or 4 condition [5,6,11]. Using the new or recurrent stage 3 or 4 condition, the sensitivity dropped to 20.9% compared to 25.2% for the clinical and immunological WHO criteria while the specificity decreased from 73.7% to 70.6%, respectively.

## Discussion

Many reports have shown that viral load is the single most important variable that should be used for long-term optimization of ART in resource-limited settings [32]. Several studies from resource-limited settings have provided evidence that supports the need for viral load monitoring of ART or at least the use of an algorithm to target viral load testing [16,17,33–35].

Our findings support others from resource-limited settings that report a poor sensitivity of WHO clinical and CD4 criteria to identify treatment failure (sensitivity 15.2–36.4%) [6,13,16,17]. We found that a predictor score with CD4 criteria performed better than WHO clinico-immunological criteria or WHO clinical staging (sensitivity 57.1% vs. 25.2% and 20.9%, respectively). The low PPV is not atypical of those previously reported [6,13]. Our predictor score includes WHO clinical staging and the diagnosis of a new condition or symptom which highlights the value of clinical judgment in diagnosing treatment failure. The AUC to detect virologic failure was calculated as 0.62 (for the score with CD4 criteria) which is less than others have reported for derivation (0.70) and validation (0.75) populations but is similar to what has been reported in routine care (0.69 95% CI 0.60–0.77) [36,37].

The sensitivity of the predictor score presented here is similar to the sensitivity of an algorithm developed in Kampala, Uganda (Se 57.1% vs. 67%) although this algorithm would result in more false positives (Sp 50.5% vs. 82%) [6]. In their analysis, virologic failure was defined as a single viral load >400 copies/ml. This has been identified by others as a possible weakness since the majority of patients who have intermediate or high viraemia achieve viral suppression at a second measurement [37–39]. Thus, a single measurement may overestimate the failure rate. In our analysis, we defined virologic failure as sustained or two consecutive HIV-RNA (≥400 copies/ml) following suppression below this level [24]. Two consecutive episodes is regarded as a more reliable definition of virologic failure [14,40].

Another strength of the predictor score is that the laboratory and clinical information was obtained from the visit preceding virologic failure (median 225 days IQR 176–255) and not at the visit, the same day blood was drawn for the viral load [31,37]. This has the additional advantage over other scoring systems in that it can target high-risk patients for interventions (e.g. intensive adherence counselling or incentivized visit attendance) prior to virologic failure. As van Griensven and colleagues highlight, the exact time points during ART when these scores should be applied, for different populations and settings, is unknown [37]. Therefore, we considered two time points, that is, 1) virologic failure in the first 18 months after ART initiation and 2) virologic failure after 18 months on ART. Although the sensitivity decreased from 67.3 to 51.8% and the specificity increased from 41.7 to 55.1%, we identified the same variables for the score in the first 18 months and after 18 months as were identified for the combined score. This suggests that the score may be applied at routine clinic visits while on first- or second-line ART (providing that the patient has been on treatment for more than six months) and combined with targeted viral load testing.

In agreement with Labhardt and colleagues who reported an increase in mortality in patients with a score ≥5, we showed an increase in mortality in those with a score ≥4 vs. <4 [31]. Those with a score ≥4 (229/1889; 12.1%) had an increased risk of mortality compared to those with a score <4 (136/1786; 7.6%) (HR 1.71; 95% CI 1.38–2.11). Discordant responses, including incomplete viral suppression in the presence of immunologic response, known to occur in resource-limited settings, may be associated with increased mortality [41].

These findings should be interpreted in light of some limitations. First, since the score is dependent on multiple study variables, the analysis is limited by missing data. As mentioned, self-reported adherence had too few observations and could not be included in the model, therefore missed medical visits or missed ARV drug pickup were used as a measure of adherence [27]. Second, the sensitivity of the predictor score with CD4 criteria performed better than the predictor score without CD4 criteria (57.1% vs. 40.9%). Although the usefulness of the predictor score would be limited by access to CD4 count measurements, Marinucci and colleagues reported that by 2010 over 60% of rural ART clinic had access to CD4 count measurements on site [28]. Therefore, the predictor score could be applicable to most resource-limited settings. Third, consistent with other studies, we did not report on individual conditions but rather grouped them into new or recurrent WHO stage III/IV condition and diagnosis of new condition or symptom [6,37]. Fourth, conditions or symptoms can be reported at scheduled and unscheduled medical visits while viral load is only measured every 6–12 months. It is therefore possible that virologic failure precedes the report of a new condition or symptom resulting in temporal ambiguity. Also, we do not distinguish between immune reconstitution inflammatory syndrome (IRIS) and opportunistic infections. Finally, as the ART treatment guidelines change to initiate patients at a CD4 threshold <350 cells/mm^3^ or <500 cells/mm^3^, it is not clear how this will affect the predictor score – a point that was highlighted by van Griensven and colleagues [37].

WHO recognizes the importance of regular monitoring, including viral load, for reinforcing adherence to ART [42,43]. This algorithm may be useful in settings, which lack the capacity to implement routine viral load testing, to discriminate between patients in need of adherence support and those that may require testing to confirm virologic failure (Figures 1 and 2). We are currently testing the scoring algorithm prospectively, in the same setting, to determine if a changing (worsening) score can predict virologic failure or perhaps identify poor adherence in patients on ART. Since monitoring strategies for resource-constrained settings should include simpler, cheaper assays or methods that can be administered by minimally trained clinic personnel, it will be interesting to see how the predictor score administered by a primary health care nurse compares to ART monitoring performed by clinicians, and used to derive the predictor score.

## Conclusions

We identified a predictor score that had an acceptable diagnostic performance – comparable to other studies – and performs better than WHO clinic-immunological criteria or WHO staging. This algorithm has a number of strengths, including virologic failure based on two consecutive viral loads, the score uses clinical and laboratory values at the visit preceding virologic failure and that the score can be applied at any point six months after the start of the treatment.

The advantage of scoring systems is that they are more flexible than WHO criteria in allowing ART providers to choose the proportion of the population that undergo targeted viral load testing (i.e. the cut-off at which to allocate viral load assays is flexible) [34]. For example, a lower cut-off (<4 vs. <3) could be used to increase sensitivity (57.1% vs. 75.7%) at the cost of relying on more viral loads. Practical application of the algorithm developed with CD4 criteria could mean that approximately 40% of true viral failure will be misclassified as viral suppression by the algorithm. Perhaps further categorizing patients into low-, medium- and high-risk for virologic failure where medium-risk patients are targeted for intensive support including more frequent monitoring or adherence counselling may help avoid late switching from a failing regimen. Importantly, as many recommend, where possible the viral load should be confirmed before switching therapy [5,6,34,44]. This avoids premature or inappropriate switching to more costly and complicated second-line regimens. Although studies indicate that the predictive value of predictor scores can be improved by adding laboratory and clinical information – to date, none have shown to be accurate enough to replace viral load testing [31].
